# Supplementary material for: Co-targeting the tumor endothelium and P-selectin-expressing glioblastoma cells leads to a remarkable therapeutic outcome
Source: eLife. 2017 Oct 4;6:e25281. doi: 10.7554/eLife.25281 (PMC5644959; doi:10.7554/eLife.25281)
Supplement: Transparent reporting form [file elife-25281-transrepform.docx]

***eLife’s* transparent reporting form**

We encourage authors to provide detailed information *within their submission* to facilitate the interpretation and replication of experiments. If you have any questions, please contact us: [editorial@elifesciences.org](mailto:editorial@elifesciences.org).

**Sample-size estimation**

- You should state whether an appropriate sample size was computed when the study was being designed
- You should state the statistical method of sample size computation and any required assumptions
- If no explicit power analysis was used, you should describe how you decided what sample (replicate) size (number) to use

Please outline where this information can be found within the submission (e.g., page numbers or figure legends), or explain why this information doesn’t apply to your submission:

*In vitro* experiments were repeated three times, with 3 technical replicates where applicable.

*In vivo* experiments evaluating the targeting capabilities of dPGS-PTX or the anti-angiogenic properties of TSP-1 PM included 2-3 mice per treatment group per each time point.

In vivo experiments evaluating the efficacy and safety of our combination treatment included 11 groups of different treatments and their relevant controls. A total of 100 mice were inoculated with glioblastoma cells intracranially. Tumor take for the GL261 murine glioblastoma model is approximately 90%, leaving an average of 8 mice per treatment group.

For blood count and biochemistry analysis, blood was drawn from 4 mice in each treatment group. However, due to an inadequate amount of blood or blood clotting, in some cases the number of mice per group was lower.

This intermediate analyses resulted in final numbers of 4-8 mice per treatment group.

This information appears in all figure legends.

As no explicit power analysis was used to determine sample size, we relied on vast previous experience of similar in vitro and in vivo methodologies.

**Replicates**

- You should report how often each experiment was performed
- You should include a definition of biological versus technical replication
- The data obtained should be provided and sufficient information should be provided to indicate the number of independent biological and/or technical replicates
- If you encountered any outliers, you should describe how these were handled
- Criteria for exclusion/inclusion of data should be clearly stated
- High-throughput sequence data should be uploaded before submission, with a private link for reviewers provided (these are available from both GEO and ArrayExpress)

Please outline where this information can be found within the submission (e.g., page numbers or figure legends), or explain why this information doesn’t apply to your submission:

Data regarding replicates and number of experimental repeats can be found in all figure legends.

**Statistical reporting**

- Statistical analysis methods should be described and justified
- Raw data should be presented in figures whenever informative to do so (typically when N per group is less than 10)
- For each experiment, you should identify the statistical tests used, exact values of N, definitions of center, methods of multiple test correction, and dispersion and precision measures (e.g., mean, median, SD, SEM, confidence intervals; and, for the major substantive results, a measure of effect size (e.g., Pearson's r, Cohen's d)
- Report exact p-values wherever possible alongside the summary statistics and 95% confidence intervals. These should be reported for all key questions and not only when the p-value is less than 0.05.

Statistical analysis methods are described in details in the materials and methods section (page 23). Exact n values and p values are specified in all figure legends for each experiment.

Please outline where this information can be found within the submission (e.g., page numbers or figure legends), or explain why this information doesn’t apply to your submission:

(For large datasets, or papers with a very large number of statistical tests, you may upload a single table file with tests, Ns, etc., with reference to page numbers in the manuscript.)

**Additional data files (“source data”)**

- We encourage you to upload relevant additional data files, such as numerical data that are represented as a graph in a figure, or as a summary table
- Where provided, these should be in the most useful format, and they can be uploaded as “Source data” files linked to a main figure or table
- Include model definition files including the full list of parameters used
- Include code used for data analysis (e.g., R, MatLab)
- Avoid stating that data files are “available upon request”

Please indicate the figures or tables for which source data files have been provided:

There are no large datasets included in this manuscript as it includes chemical synthesis and physico-chemical characterization.

The data of the biological in vitro and in vivo efficacy of the compounds synthesized in this study are attached herein.
